# PHYSICAL FRAILTY, COGNITIVE FRAILTY, AND THE RISK OF DEMENTIA IN THE GAIT AND BRAIN STUDY

**DOI:** 10.1093/geroni/igz038.1466

**Published:** 2019-11-08

**Authors:** Manuel Montero-Odasso, Yanina S Adamson, Susan Muir-Hunter, Tim Doherty, Alvaro Casas-Herrero, Richard Camicioli, Jennie Wells, Mark Speechley

**Affiliations:** 1 University of Western Ontario, London, Ontario, Canada; 2 Parkwood Institute, University of Western Ontario, London, Ontario, Canada; 3 Parkwood Hospital, London, Ontario, Canada; 4 F.E.A Geriatría Complejo Hospitalario, Pamplona, Navarra, Spain; 5 WC Mackenzie Health Sciences Centre, Edmonton, Alberta, Canada

## Abstract

Cognitive-frailty has been proposed as a distinctive entity which preludes dementia. We examined the relationship between physical frailty, cognitive status, and gait performance as predictors of cognitive decline and incident dementia. Using a cohort study of 252 community older adults free of dementia at baseline, we found that participants with frailty had a higher prevalence of cognitive impairment (77%) compared to those without (54%, p=0.02) but the risk of progression to dementia was not significant. Adding cognitive impairment to the frailty phenotype (cognitive-frailty) predicted further cognitive impairment and progression to dementia. However, when the slow gait component of frailty was combined with baseline cognitive impairment, it showed the highest risk of progression to dementia (HR: 35.9; 95%CI: 4.0–319.2; p= 0.001). Frailty and Cognitive impairment are common and co-exist in the same individuals. However, slowing gait seems to be the frailty component driving the association with future dementia.

